# Flexibility and resilience in equity-centered research: lessons learned conducting a randomized controlled trial of a family-based substance use prevention program for American Indian families

**DOI:** 10.3389/fpubh.2024.1459294

**Published:** 2024-12-09

**Authors:** Nancy L. Asdigian, Nicole R. Tuitt, Rhonda Dick, Monica Fitzgerald, Tracy Zacher, Lisa Bear Robe, Carly Shangreau, Raeann Vossberg, Candace Fleming, Nancy Rumbaugh Whitesell

**Affiliations:** ^1^Centers for American Indian & Alaska Native Health, Colorado School of Public Health, University of Colorado Anschutz Medical Campus, Aurora, CO, United States; ^2^Missouri Breaks Industries Research, Inc., Manderson, SD, United States

**Keywords:** American Indian, equity-centered research, Indigenous methodology, substance use prevention, academic-community partnership

## Abstract

Meaningful and effective community engagement lies at the core of equity-centered research, which is a powerful tool for addressing health disparities in American Indian (AI) communities. It is essential for centering Indigenous wisdom as a source of solutions and disrupting Western-centric perspectives and inequitable and exclusionary research practices. This paper reports on lessons learned implementing an effectiveness trial of the Thiwáhe Glúwašʼakapi program (TG) program (translated as “sacred home in which families are made strong”)—a family-based substance use prevention program—in a post-pandemic era with an American Indian reservation community that has confronted extreme challenges. We describe lessons in six areas (community engagement, study design, community workforce, participant engagement, retention, and data collection) that illustrate how conventional Western research practices were adapted in order to conduct authentic, equity-centered research. Key principles gleaned from those lessons are also discussed, including: (a) honoring partnerships and making study decisions collaboratively, (b) considering the implications of decisions for both the scientific value of the study and the community, (c) considering the health and well-being of community staff, (d) being flexible and responsive to changing needs, and (e) approaching work with communities through a strengths-based frame. Insight into the challenges encountered and the solutions developed in alignment with community partners and Indigenous wisdom may strengthen the work of other academic-community partnerships endeavoring to bring culturally relevant, evidence-informed prevention programming to Indigenous communities.

## Introduction

Meaningful and effective community engagement lies at the core of equity-centered research, which is a powerful tool for addressing health disparities in American Indian (AI) communities (1–7). It centers the unique lived experiences and perspectives of community partners, which creates a comprehensive understanding of the factors that shape health outcomes and informs decisions about relevant and impactful interventions (8–10). True engagement requires researchers to actively listen throughout all phases of a research project and adapt their methods to be responsive to the input of community partners and to the historical, cultural, and community context in which the research is being conducted. It is essential for centering Indigenous wisdom as a source of solutions and disrupting Western-centric perspectives and inequitable and exclusionary research practices (1).

Western methodologies center individualistic assumptions and value quantifiable methodologies that often lack historical perspective and context and are incongruent with many Indigenous frameworks (11–13). In contrast, Indigenous methodologies center teachings and understandings that include spirituality, history, culture, and holistic and relational ways of knowing and being (10–16). They rest on foundational principles that knowledge is inherently: (a) place-based, context-specific, and positional rather than consisting of singular, universal truths; (b) experiential and practical rather than conceptual or abstract; (c) holistic and relational rather than compartmentalized and disconnected; and (d) framed through a strengths-based rather than a deficit-focused perspective (17–20). They are driven by Indigenous priorities and used in service of advancing their own communities; use a reflexive process; are decolonizing and reflect self-determination and sovereignty; center the research process rather than only the outcomes of research; integrate responsibility, reciprocity, and knowledge sharing; and respect and privilege Indigenous world views, epistemologies, and ethics throughout all phases of a research project (15, 18, 19, 21).

An increasing focus on equity-centered research has highlighted the misalignment of Western methodologies with many contexts and cultures and has paved the way for a paradigm shift that privileges Indigenous methodologies or, at a minimum, demands their integration with Western approaches. “Two-Eyed Seeing” is an emerging conceptual framework that acknowledges the strengths of both Indigenous and Western world views and highlights the value of using components of each perspective when appropriate (3). Incorporating Indigenous methodologies has historically presented challenges in the context of federal research funding and narrow standards of methodological rigor, although initiatives over the last decade have increasingly centered Indigenous approaches as critical to rigor rather than antithetical to it (22–24).

With funding from the Intervention Research to Improve Native American Health (IRINAH) initiative (15), we partnered with a Northern Plains AI reservation community to address early substance use in their community. This paper reports on lessons learned implementing an effectiveness trial of a family-based substance use prevention program that emerged from this partnership and was adapted and co-created to fit the cultural context of the community (25, 26). In doing so, it highlights key challenges that needed to be addressed when integrating Indigenous perspectives and centering community priorities into a Western research approach to evaluating intervention effectiveness.

In telling the story of this work, we discuss lessons learned about conducting research in a post-pandemic era with an AI reservation community that surmounted significant COVID-19 pandemic losses and continues to confront challenges such as extreme weather, workforce shortages, family trauma, and substantial poverty—social outcomes that reflect the legacy of settler colonialism (16). Insight into those challenges and the solutions developed in alignment with community partners and Indigenous wisdom, may strengthen the work of other academic-community partnerships endeavoring to bring culturally relevant, evidence-informed prevention programming to Indigenous communities.

## Context: study setting and target population

The current project is part of a 25 year collaboration between academic researchers (authors NLA, NRT, RD, MF, CF, RV, NRW) from the Centers for American Indian and Alaska Native Health at the University of Colorado Anschutz Medical Campus and partners from a Northern Plains AI reservation community (authors CS, TZ, LBR) to understand substance use and mental health epidemiology and etiology. The academic researchers approach their work from an equity-centered and strengths-based perspective. One of them is Indigenous; all have extensive experience conducting research in partnership with Indigenous communities and, in particular, in the community that was a part of this study. All project partners are experienced Indigenous researchers who live and work in the participating community and all have worked with academic researchers from CAIANH and other universities for many years. The current academic and community partner team have successfully worked together for approximately 10 years.

Most proximal to this work was a secondary analysis of data collected from the Wiconi Teca Waste project in 2014 on patterns of substance use initiation and early use among middle school students in the community (27). That analysis was conducted in partnership with a nine-person Community Advisory Board (CAB) of elders, health care providers, substance use experts, educators, and other tribal community members. Recognizing the community needs identified through this research, the CAB urged us to find effective prevention approaches that aligned with and incorporated community and cultural strengths. They guided us to select The Iowa Strengthening Families Program for Parents and Youth 10–14 (ISFP) (28) as the foundation for a program into which a series of community-informed cultural and contextual adaptations were integrated and evaluated (25, 26). That work gave rise to the Thiwáhe Glúwašʼakapi program (TG) program, translated as “sacred home in which families are made strong,” which showed promising short-term effects on proximal outcomes among both youth and adult^1^ participants (29).

The current phase of this research involves an Individually Randomized Group Treatment Trial (IRGT) evaluating the effectiveness of the optimized TG program over a multi-year follow up period. The study design called for randomly assigning schools on the reservation to four cohorts, stratified by region, over a two-year implementation period. It also involved working with community-based research staff to recruit families from each site and randomly assign them (based on a 50/50 split) to either the TG program or a comparison nutrition text-messaging intervention (Woyute Waśte, “good food”). Up to two youth and one caregiving adult from each enrolled family would complete a baseline survey at enrollment and an immediate post-program survey approximately 8 weeks later (1 week after the completion of the seven-week TG program). Participants would also be invited to complete follow-up surveys every 6 months after program completion for up to 3 years.

Although our original focus during intervention development was on youth substance use, we expanded outcomes in the RCT to include youth suicide risk. This change was based on feedback from parents and advisors about community priorities, combined with evidence that many of the factors addressed in the TG program (e.g., family connectedness, strengthening of cultural ties) are protective against both substance use and suicide risk (30–32). We also broadened evaluation outcomes to include caregivers’ own substance use behavior, based on findings from the optimization trial (26) that a sizeable minority of participating adults reported problematic substance use. Information about program impact on this constellation of clustered risk behaviors will be critical to informing community decisions about how to invest limited prevention resources in the face of complex challenges to the health and well-being of the community (33–36).

If evidence from the RCT demonstrates effectiveness, the vision is to sustainably integrate TG into existing institutional structures (e.g., schools, existing family support programs) in order to change the context of early substance use in this community. Another goal is to expand TG to other Indigenous communities, providing guidance on adaptations for specific cultural contexts.

## Challenges and lessons learned

The RCT of the TG program was paused for almost 2 years shortly after it was launched in February 2020, just prior to the COVID-19 pandemic lockdown. When we were able to restart in the spring of 2022, we encountered many challenges, some of them lingering effects of the pandemic and others ongoing in the context of this remote AI reservation community (e.g., poverty and unemployment due to lack of opportunities; significant health disparities stemming from inadequate access to health care; elevated levels of trauma as a legacy of colonial oppression). As we moved through this work, we learned important lessons about the need to adapt conventional Western research approaches to be responsive to community context. We distilled key lessons in six areas—community engagement, study design, community workforce, participant engagement, retention, and data collection.

### Community engagement

As in earlier phases of this research program, university and community research partners worked closely together in the RCT. It would be inappropriate and likely impossible to do this work otherwise. Community priorities drive research agendas and expertise provides cultural context and informs all aspects of research including study design; participant recruitment; program implementation; and data collection, analysis, and interpretation. University partners bring research skills, resources, and infrastructure to support the work (3, 37).

In response to pressing community needs and concerns, we paused all research activities at the onset of the COVID-19 pandemic, just after we enrolled the first cohort and collected baseline data, but before program implementation. Out of concern for the safety of participants and staff, and in adherence with Tribal restrictions on travel and in-person gatherings, we were unable to resume in-person activities for 2 years. During this period, we met weekly with community project staff to refine strategies for family recruitment and retention, improve study measures and data collection protocols, and reinforce implementation and evaluation trainings. We instituted monthly Community of Learning meetings to support bidirectional learning and further enhance capacity among both community and university staff. At the urging of our community partners, we worked with tribal language experts to translate the family creeds that are promoted in the TG program, a process that greatly enhanced the cultural alignment of those creeds. We attended virtual school board meetings; checked in with schools; and participated in virtual tribal research review meetings to provide study updates, discuss protocol amendments, and get updated guidance on in-person activities. We also recruited cultural leaders and staff from schools and other community organizations to participate in an Implementation Action Council that would help plan for sustainable implementation in the community. Taken together, these activities strengthened study infrastructure, built capacity among community partners, improved the intervention, and created model procedures that can be used in future research with remote reservation communities.

### Study design

From the outset, this study required creativity to balance community priorities and scientific principles. Most Western scientific models for gathering data on long-term intervention effects require randomization to support the internal validity of study findings (38–40). Across all RCT variations, some participants, groups, or communities get the intervention immediately, others get it later or never get it (39, 40). This sort of allocation of intervention resources is regarded as unethical in many Indigenous communities, particularly where the intervention could benefit those with urgent needs (27). As such, we had to balance the scientific value of the RCT with guidance from our community partners to ensure that all families in the study receive something of value. Thus, the design we chose included a comparison group that received a culturally adapted text-messaging program focused on healthy eating and active living (Woyute Waśte [WW]). The comparison addressed a priority community need while also being cost-effective for the study and allowing us to focus resources on the evaluation of effectiveness of TG.

Despite the extensive work and joint decision making that went into the selection of the WW program for the RCT comparison arm, the difference between the study arms created a barrier to recruitment. The possibility of being randomized to the WW program was off-putting to some families. Community partners reported that some families refused to participate when they found out they would not have a choice of programs. Others chose to drop out of TG if the family of a close friend or relative was randomized into WW, because they intended to participate in the TG program together. In addition, by virtue of its lower intensity, participation in the WW program was associated with fewer incentives (i.e., no incentives for session attendance), which may have further dampened enthusiasm. Moreover, in the early months of the pandemic, we decided to transform WW into a fully virtual format to reduce participant risk, eliminating an in-person family session designed to kick off the program. Although this change was a reasonable adaptation to the pandemic, it may have further complicated recruitment challenges. Finally, because recruitment numbers fell below expectations, intervention groups were smaller than intended, which adversely impacted the way families experienced the TG program. To maximize the number of families randomized into TG, we adjusted our allocation strategy from 50/50 to 60/40 in favor of the TG program. This also increased the likelihood that families would be assigned to the program most preferred, which was more acceptable in the community.

Continued struggles with recruitment and the recognition that, despite our best efforts, our quantitative sample would be much smaller than we planned, led us to adopt a mixed methods approach to triangulate effectiveness findings. Qualitative approaches align well with Indigenous methods of knowing and provide a rigorous complement to quantitative methods without being dependent on large samples for meaningful analysis (19, 41, 42). We interviewed adults and youth TG participants about their perceptions of how the program affected their caregiving behaviors, family interactions, substance use, and emotional well-being outcomes. We also interviewed families who were randomized into TG but either failed to attend any TG sessions or attended very few sessions so we could better understand barriers to engagement. Analyzing the quantitative and qualitative data in tandem will allow us to leverage the small sample size and maximize what can be learned from this study.

### Community workforce

Research professionals who work in reservation communities provide invaluable support through their knowledge and understanding of the community context and the trust that community members have for them. TG is designed to be delivered by trained community facilitators, and community staff are needed to recruit, enroll, and collect data from study participants. This project was staffed through a partnership with a Native-run health research organization with an office in the reservation community. They employ skilled and experienced community staff to provide research support to various universities and research organizations. Some of the staff who worked on this project were involved in the previous TG program development and optimization trial, both as program facilitators and data collectors.

Despite the expertise and experience of the local workforce, staffing was a challenge in this remote reservation community. Hiring and retaining qualified staff were issues when the university maintained a community field office during the optimization phase of this work (26) and continued to be so during the RCT phase. Limited project budgets exacerbate those issues by requiring experienced staff to be shared across projects. During the pandemic when we were unable to engage in in-person activities, project staff shifted to other projects rather than remain idle. Those shifts sometimes made it difficult for staff to return to this study when we were able to resume implementation. Some staff who were trained for TG were no longer available to facilitate the program, and others had to be trained to replace them.

Staff turnover was also an issue, especially among staff who delivered the TG program to families. The work schedule is demanding, especially for staff who have families and/or are in school, as it involves facilitating program sessions several nights during the week, combined with hosting recruitment and data collection events in the evenings and on weekends. The typical challenges working with families were amplified by the high levels of stress and trauma some families experienced as a result of the COVID-19 pandemic. Facilitators were experiencing secondary trauma at higher levels after the pandemic than they had in pre-pandemic phases of this study. Staff turnover caused delays and understaffing for some project activities, put increased pressure on the remaining staff who were called upon to assume additional responsibilities, and necessitated repeated training of new staff.

Moreover, because our goal was to understand program impacts over an extended time period, including the transition from middle to high school, our design involved up to six waves of post-program assessments over a three-year period for youth and adult participants. This ambitious data collection protocol required us to develop sophisticated participant tracking and data collection systems and to train community research staff on those protocols (43). Complex data collection tasks such as these require vastly different skills (i.e., administering informed consent, managing data collection systems, tracking survey completion) than facilitating program sessions (i.e., building relationships with families, delivering curricula, facilitating discussions, managing group dynamics). It proved difficult for staff who were hired primarily based on their ability to engage with families and facilitate the TG program to also support the intensive data collection needs of the study.

In response to these challenges, we learned the value of working in close coordination with our community research partners to address new and ongoing staff training needs and create efficient training mechanisms such as recorded trainings accompanied by live support and a certified TG trainer in the community to support facilitators. Study-related activities involving community staff were planned and scheduled to create an acceptable work-life balance. In addition, a Native clinical psychologist who has worked for many years with this community was hired to meet weekly with program facilitators and provide a form of reflective supervision to help them process their experiences with families and their reactions to families’ trauma (44). Weekly group reflective supervision was instituted during program implementation, with optional individual sessions for facilitators as needed. Facilitators also received direct training in trauma informed care, to help them develop strategies for interacting with families in this challenging context.

### Participant engagement

We encountered challenges in three areas related to participant engagement—eligibility, recruitment, and retention.

#### Eligibility

From the outset, we worked closely with our community research partners to determine appropriate eligibility criteria for study participants. Resource constraints and data collection considerations dictated that we could only enroll up to two youth and one adult per family as *research participants* who would earn incentives for survey completion (both study arms) and session attendance (TG arm only). However, our partners told us that, given the high prevalence of multigenerational households and extended family caregiving in the community, it was imperative to allow other family members to participate in TG sessions with enrolled family members. We thus invited additional youth (aged 10–13) and adult family members to join as *program only* participants; they were included in family meals and participated in intervention activities but did not complete surveys or receive compensation for participation. Although this approach addressed the need to include extended family members, it did create equity issues when compensation was available for some family members but not others.

#### Recruitment

Lingering effects of the COVID-19 pandemic dramatically limited in-person opportunities for recruitment (e.g., pow-wows, parents’ nights at schools, sporting events). Obtaining the support of schools in recruiting eligible families was more difficult than before the pandemic. Because they were overstretched with other priorities related to pandemic disruptions in learning, being available to coordinate recruitment logistics with our team was not always possible. Moreover, all in-person recruitment efforts were limited by seasonal weather constraints, including record levels of snowfall on the reservation.

In this context, we had to rely heavily on remote recruitment efforts such as posting flyers at stores, churches, and other community venues with a QR code to collect contact information of interested participants; sending flyers home with students; radio advertisements; and posts on Facebook that included raffles for prizes. These strategies were dramatically less effective than in-person efforts.

These challenges underscored the value of conducting in-person recruitment whenever possible. School functions such as parent-teacher conferences, parent/grandparent nights, back-to-school events, school enrollment days, and basketball games have been particularly successful avenues for recruitment, especially when we were able to set up an information table and offer families token incentives (e.g., gift bags, magnets) or meals to learn more about the study. We experimented with door-to-door recruitment and found mixed success. We were able to enroll families through this strategy, but many showed weak commitments and did not consistently attend program sessions once enrolled. Families may have found it hard to say no to a friendly program recruiter at their door even if they were not sure they could participate. Overall, families were less likely to enroll and less likely to stay engaged when we resumed implementation in 2022 than they had been before the pandemic. Conversations with families about barriers reflected ongoing pandemic-related fears, trauma associated with the loss of family members, and post-pandemic inflation pressures as challenges.

Another recruitment challenge we encountered when we resumed project activities in 2022 was related to the COVID-19 vaccine requirement. Following guidance from the Tribe, we required that all participating family members and staff be vaccinated (or have a verified exemption). This was a barrier for some families who were interested but had unvaccinated family members or were unable to locate or otherwise share their vaccination cards. We were able to relax the vaccination requirement in the fall of 2023, per CDC and Tribal guidance, and saw recruitment increase significantly.

Our post-pandemic recruitment efforts clearly highlighted how time- and resource-intensive it is to recruit families to participate in research activities that involve committing to several weeks of program sessions. Families have multiple competing priorities for their time and making space for a 7-week program is often not their most pressing concern.

Several key lessons emerged from these experiences: (a) staff have to devote sufficient time to recruitment efforts, (b) recruitment efforts need to be conducted well in advance of the start of program activities so that families can reserve space in their calendars for participation, (c) conversations with families about recruitment should ensure that families understand what participation involves and that they do not feel pressured to sign up if it is not a good fit for them; and (d), recruitment efforts are most successful when they build relationships between program staff and potential participants, as these connections are a foundation for future engagement and retention.

### Retention

Our experience also demonstrated that successful recruitment efforts do not necessarily translate into successful engagement and retention once implementation is underway. TG program attendance varied significantly between communities and individual families; some families attended no sessions while others attended most or all of the sessions. Across the four cohorts, the average attendance rate for participants randomized into TG was 42.6% (i.e., about 3 out of the 7 sessions). This is likely due to a variety of barriers, including transportation, childcare needs, competing priorities for busy families, and residual trauma from the COVID-19 pandemic. Different strategies are needed to ensure that enrolled participants remain engaged throughout program sessions and are retained afterwards during post-program data collection. Some of these strategies were utilized before the pandemic, but to a lesser extent; others were new strategies used when we returned to in-person implementation.

#### Logistical support for families

First, with the help of our community research partners, we took steps to reduce barriers to family participation, including increasing the provision of childcare for younger children so that parents and other caregivers could participate with their 10- to 13-year-old youth(s). We provided family meals at the beginning of each TG session, which has important cultural significance in this and other Indigenous communities and supports relationship building among families and between program facilitators and families. We also provided transportation for families who could not otherwise attend program sessions in this remote reservation community. For families who had reliable transportation, we provided gas cards to ease the economic burden of attending sessions.

#### Emotional support for families and facilitators

The most striking impact of the COVID-19 pandemic on implementation activities was the trauma it created for both families and program facilitators. The pandemic impacted this reservation community deeply. Most—if not all families—lost family members to the pandemic. Many elders, Native language speakers, spiritual leaders, and cultural teachers were lost, and traditional ways of honoring those lives could not be followed. Cancelations of community cultural events cut people off from support sources when they were sorely needed. While these kinds of experiences were shared across many communities around the country and the world, the extent of the impact was particularly heavy in this and other American Indian communities in the United States (45, 46).

#### Pandemic protocol monitoring

We encountered other challenges directly related to the pandemic. Facilitators had to implement and enforce public health safety practices, including disinfecting surfaces before and after sessions, reminding families of mask requirements, and promoting social distancing. These roles further complicated their relationships with families, which likely made it more difficult to build the kind of trusting relationships with both adults and youth that encourage families to keep coming back week after week.

### Data collection

Data collection in rural reservation communities presents a unique set of challenges under the best of circumstances. Data collection in the post-pandemic context was particularly tricky. We quickly realized that we needed to modify our data collection systems and procedures to address emerging challenges. When possible, surveys were administered online to reduce the need for in-person interaction. This was particularly important in addressing COVID-19 restrictions, travel restrictions due to weather conditions, and transportation barriers. However, online survey administration also laid bare striking inequities related to the digital divide, including the lack of technical infrastructure and limited access to Wi-Fi within rural reservation communities. When necessary, community research staff took devices (tablets for online survey access) and/or materials (paper surveys) to participants’ homes to facilitate data collection. For participants without access to home Wi-Fi at home, staff would either meet them at or transport them to local community venues (e.g., churches, community centers, staff office, schools, etc.) that had public Wi-Fi access.

Online survey administration also brought with it security and privacy challenges for families, particularly because it is common for cell phones to be shared among family members in this community. We were concerned about youth being reluctant to answer survey questions honestly if they felt their caregiver might be able to access their answers on a common device. We likewise needed to reassure participants that community-based staff, with whom many live and work, were unable to access any of their survey data. To ensure data security and privacy, we required all youth and adult participants to create survey passwords and instituted a secure protocol for password recovery exclusively by university staff.

Finally, literacy in the community was also an issue and COVID-19-related fluctuations in education exacerbated the issue (29). Post-pandemic reading delays among the 10–13 year old youth enrolled in the program were striking. The disruption of in-person learning was particularly difficult in remote areas of the reservation where internet access is unreliable and, as a result, many youth fell further behind than their peers in other areas of the country. We accommodated literacy needs by providing youth with slides that narrated the assent form and offering that option to adults as an alternative to reading written consent and parent permission forms. We also provided both youth and adults with an on-demand option in the online survey for computer-generated audio to read any of the survey questions or instructions.

## Discussion

As we encountered the challenges described above, we adapted and developed solutions by adhering to a set of principles that supported the integrity of the study while allowing us to be flexible and responsive to community contexts and needs.

### Principle 1: honor partnership and make decisions collaboratively

Equitable engagement of community partners is critical to our work given the history of harm and maltreatment that AI populations have been subjected to by external researchers (47, 48). To prevent further harm, our research team centered the perspectives, knowledge, resources, strengths, and skills of community partners to implement the study. At each pivot point we encountered, we engaged input from all study partners, including researchers in the university and in the community. Collaborative decision making respected the insights and experiences of all team members, leveraged them to ensure the integrity and utility of the data we could gather, and aligned with the relational focus of Indigenous methodologies (3, 17–19) and principles of Indigenous Community Based Participatory Research (ICBPR) (5).

### Principle 2: consider the implications for the scientific value of the study and to the community

We considered the repercussions of each proposed change to study methodology for both the scientific rigor of the study and the needs of the community. Each time we encountered a barrier, researchers and community partners jointly identified and discussed options for moving forward. We relied on the diverse expertise in our team to help us think through the implications of each option for our sample, our data, our analyses, and equally important, the community. In line with key tenets of Indigenous methodologies that center community needs and prioritize place-based and experiential knowledge (17, 19, 37) we were intentional about balancing best scientific practices with community needs and priorities.

### Principle 3: consider the implications for study staff

We also prioritized relationships by attending carefully to the implications of methodological decisions for study staff (5, 17–19). Community staff carried a heavy load, worked under exceedingly difficult conditions (e.g., pandemic protocols) and engaged with families experiencing elevated levels of stress. We remained mindful of how our decisions would impact their workload, relationships in the community, and personal wellbeing. We also developed processes for ensuring that the facilitators received the support they needed to have a sense of physical, psychological, and social safety.

### Principle 4: remain flexible and responsive to changing needs

Although we were strongly committed to ensuring a rigorous study design throughout, we were also keenly aware of the need to be responsive to the community and historical context of the study—i.e., conducting this study in a remote reservation community in the wake of the pandemic. There were times we had to accept that favored approaches would be impossible and thus shift our focus to making the most of the other options available. This required a place-based, experiential orientation involving flexible thinking about approaches that provide the best data and support the most meaningful analyses within the constraints we encountered (5, 17, 19, 20).

### Principle 5: approach this work through a strengths-based frame

We recognized early on that the study would not go as planned and we would not be able to collect all the data we intended. Rather than being immobilized by this, we adopted a strengths-based perspective and took the time to listen to the wisdom of our community partners about what we could do instead. The collaboration and guidance of community staff and advisors gave rise to important lessons about how to conduct this study in a good way—lessons that can inform equity-centered research with Indigenous communities in the future.

### Summary

Despite the challenges we encountered implementating a large scale RCT in a post-pandemic environment and being responsive to Indigenous perspectives and community priorities, the value of continuing with the study was never in doubt. Progress was not linear, and study activities did not unfold as we had envisioned. Nonetheless, our experience provided rich opportunities for rethinking the way we approach research in partnership with community. A commitment to flexibility allowed us to enroll families, randomize them to study arms, and collect data on their experiences. Families that were able to participate in TG responded positively to the opportunity to meet with other families, share experiences, and learn strategies for strengthening their families and protecting their youth. They have remained engaged in follow-up data collection. These responses inspired our team to find creative solutions to the challenges we continue to encounter, knowing that the data these families share will be invaluable in helping us understand the value of the TG program.

## Data Availability

The original contributions presented in the study are included in the article/supplementary material, further inquiries can be directed to the corresponding author.

## Footnotes

^1^Any adult who played a significant role in caregiving a youth was able to participate in the TG program with that youth, including stepparents, grandparents, aunts and uncles, older siblings, cousins, and other adult guardians. Parent is used throughout this paper to refer to any caregiving adult who participated in TG with a youth.
